# Investigating the impact of a 20 miles per hour speed limit intervention on road traffic collisions, casualties, speed and volume in Belfast, UK: 3 year follow-up outcomes of a natural experiment

**DOI:** 10.1136/jech-2022-219729

**Published:** 2022-11-15

**Authors:** Ruth F Hunter, Claire L Cleland, John Busby, Glenna Nightingale, Frank Kee, Andrew James Williams, Paul Kelly, Michael P Kelly, Karen Milton, Kelly Kokka, Ruth Jepson

**Affiliations:** 1 Centre for Public Health, Queen's University Belfast, Belfast, UK; 2 Scottish Collaboration for Public Health Research and Policy, University of Edinburgh, Edinburgh, Scotland, UK; 3 School of Medicine, University of St Andrews, St Andrews, Scotland, UK; 4 Physical Activity for Health Research Centre (PHARC), Institute for Sport, Physical Education and Health Sciences, University of Edinburgh, Edinburgh, Scotland, UK; 5 Department of Public Health and Primary Care, University of Cambridge, Cambridge, England, UK; 6 Norwich Medical School, University of East Anglia, Norwich, Norfolk, England, UK; 7 MRC Epidemiology Unit, University of Cambridge, Cambridge, UK

**Keywords:** PUBLIC HEALTH, EPIDEMIOLOGY, PREVENTION

## Abstract

**Background:**

Evidence regarding the effectiveness of 20 miles per hour (mph) speed limit interventions is limited, and rarely have long-term outcomes been assessed. We investigate the effect of a 20 mph speed limit intervention on road traffic collisions, casualties, speed and volume at 1 and 3 years post-implementation.

**Methods:**

An observational, repeated cross-sectional design was implemented, using routinely collected data for road traffic collisions, casualties, speed and volume. We evaluated difference-in-differences in collisions and casualties (intervention vs control) across three different time series and traffic speed and volume pre-implementation, at 1 and 3 years post-implementation.

**Results:**

Small reductions in road traffic collisions were observed at year 1 (3%; p=0.82) and year 3 post-implementation (15%; p=0.31) at the intervention site. Difference-in-differences analyses showed no statistically significant differences between the intervention and control sites over time for road traffic collisions. There were 16% (p=0.18) and 22% (p=0.06) reductions in casualty rates at years 1 and 3 post-implementation, respectively, at the intervention site. Results showed little change in mean traffic speed at year 1 (0.2 mph, 95% CI −0.3 to 2.4, p=0.14) and year 3 post-implementation (0.8, 95% CI −1.5 to 2.5, p=0.17). For traffic volume, a decrease in 57 vehicles per week was observed at year 1 (95% CI –162 to −14, p<0.00) and 71 vehicles at year 3 (95% CI −213 to 1, p=0.05) post-implementation.

**Conclusion:**

A 20 mph speed limit intervention implemented at city centre scale had little impact on long-term outcomes including road traffic collisions, casualties and speed, except for a reduction in traffic volume. Policymakers considering implementing 20 mph speed limit interventions should consider the fidelity, context and scale of implementation.

**WHAT IS ALREADY KNOWN ON THIS TOPIC**

- Evidence on the effectiveness of 20 miles per hour (mph) speed limit interventions are limited, with previous studies showing mixed results. Long-term outcomes have rarely been assessed.

**WHAT THIS STUDY ADDS**

- A 20 mph speed limit intervention implemented at city centre scale had little impact on short- or long-term outcomes for road traffic collisions, casualties and speed.

**HOW THIS STUDY MIGHT AFFECT RESEARCH, PRACTICE OR POLICY**

- Policymakers considering implementing 20 mph speed limit interventions should consider the fidelity, context and scale of implementation.

## Introduction

Traffic speed is an important determinant of population health,^1^ with road traffic injuries noted as one of the leading causes of preventable death globally.^2^ Approximately 50% of deaths worldwide are of pedestrians, cyclists and motorcyclists.^3^ Interventions to reduce road traffic speed to 20 miles per hour (mph) (or the equivalent 30 km per hour) have become increasingly popular in the UK and parts of Europe. Two intervention approaches currently exist to reduce traffic speed, both involving legislation, awareness/education campaigns, signage and enforcement: 20 mph ‘zones’ involve physical traffic calming measures (eg, road narrowing, speed bumps, central islands), whereas 20 mph ‘limits’ do not involve physical calming measures but use road markings instead.

The main reasons for reducing traffic speed are to lessen the likelihood of a road traffic collision (ie, an ‘incident’ involving a person and at least one road vehicle) and to reduce the severity of road traffic casualties (ie, when a person(s) is killed or injured as the result of a collision).^4 5^ The theory behind introducing speed limits is that enforcement, signage and education can improve driver compliance and reduce their average speed.^6^ Research indicates that if a pedestrian is struck by a vehicle at 24 mph, they have a 10% risk of dying.^7^ This increases to 25% at 32 mph, and 50% at 41 mph.^7^ A reduction in speed of as little as 1 mph is associated with a reduction in casualties of up to 6%.^7^ Exceeding speed limits causes 5% of all collisions and 15% of fatal collisions.^8^ At traffic speeds between 30–40 mph, the risk of pedestrian fatalities are 3.5–5.5 times more likely compared with slower speeds at 20–30 mph.^9^

Transport interventions, such as 20 mph speed limits, are popular; due to the connected and interdependent nature of transport and health, 20 mph speed limits have the potential to generate wider public health co-benefits.^10^ For example, 20 mph speed limit interventions can facilitate a smoother traffic flow and reduce traffic congestion.^11^ Consequently, smoother traffic flow has the potential to reduce fuel consumption and decrease air pollution.^11^ Other purported benefits include increased physical activity and improved liveability due to increased perceptions of safety. However, in spite of these potential public health benefits and co-benefits, public opinion on 20 mph speed limits is mixed, with some arguing that lowering traffic speed will lead to increased congestion and consequently increased air pollution.^12^ In addition, non-compliance is an issue,^12^ and the evidence base for 20 mph limits (ie, those that do not involve chicanes or speed humps but instead are primarily signage based) is inconclusive.^10^

An umbrella review of previous research concluded that 20 mph schemes can reduce collisions, injuries and traffic volume, and improve perceptions of safety, while being cost effective.^13^ However, this review did not distinguish between the impact of 20 mph ‘zones’ and 20 mph ‘limits’, and the outcomes were limited to collisions, injuries, traffic speed and volume.^13^ A more recent review by Cleland and colleagues^10^ found that 20 mph ‘zones’ are effective in reducing the number and severity of collisions and casualties. However, there was insufficient evidence to draw robust conclusions on the effect of ‘zones’ on liveability, inequalities and air quality, or the effect of ‘limits’ on these public health outcomes.^10^ The review also highlighted the need for future research to provide robust findings from high quality controlled long-term evaluations.^10^

There is a clear need to develop further the evidence base for 20 mph speed limit interventions that investigate a range of public health outcomes, and over longer timeframes. The implementation of a 20 mph speed limit intervention in Belfast city centre in 2016 afforded an opportunity for such a natural experiment.

The aim of this study was to investigate the effect of the Belfast city centre 20 mph speed limit intervention on road traffic collisions, casualties, speed and volume at 1 year and 3 years post-implementation. Objectives included:

1. To investigate the change in the rate and severity of road traffic collisions and casualties at 1 year and 3 years post-implementation
2. To investigate the change in traffic speed 1 year and 3 years post-implementation
3. To investigate the change in traffic volume 1 year and 3 years post-implementation.

## Methods

### Intervention description

The 20 mph speed limit intervention was implemented in Belfast, UK in February 2016. In total, 76 streets in the city centre were assigned a speed limit of 20 mph which was operational 24 hours a day, 7 days a week, with an estimated implementation cost of £10 000. None of the streets in the intervention area had a pre-existing 20 mph speed limit. The city centre is largely a commercial area, with little residential housing, but with high levels of pedestrian movement, cycle activity and bus facilities. City centre street widths vary little but have varying levels of on/off-street parking and pedestrian crossings and they are surrounded by a network of 30 mph and 40 mph streets.

The intervention consisted of four main elements:

- legislation (ie, Speed Limit Order)
- awareness and education campaigns (ie, a programme of awareness raising and education to publicise and support the introduction of the 20 mph speed limit intervention, to explain the benefits of lower speeds and ensure a smooth transition process)
- 20 mph signage and road markings (ie, 20 mph road markings and traffic signs installed at the places where the speed limit changes, and smaller ‘20’ repeater signs placed at regular intervals)
- enforcement (ie, warnings and issuing of speeding tickets; warnings were favoured early in the implementation phase, replaced by speeding tickets in later phases).

### Study design

We used a controlled before–after study design, using routinely collected data. As collisions and casualties data were collected across the whole of Northern Ireland it was possible to identify control areas (see Statistical analyses below). The 20 mph speed limit intervention in Belfast was implemented across streets in 10 Small Areas (11 Output Areas), and it was possible to identify matching control areas using appropriate census variables. The control areas were matched using the separate domains for the Northern Ireland Multiple Deprivation Measure (2017),^14^ the urban–rural classification of the area and population density. Geographic Information Systems (GIS) was used to allocate deprivation, population and rurality data to the implementation areas using administrative geographies. As the intervention area included more than one Output (or Small Area) the mean, median or mode of each of the matching variables was used depending on the nature and distribution of the variable. This enabled an evaluation of collisions and casualties to be compared against secular trends from matched areas (difference-in-differences).

For road traffic speed and volume, we used an observational, repeated cross-sectional, pre-post quasi-experiment design,^15^ using routinely collected data. Analyses of repeated cross-sections of data for traffic speed and traffic volume were conducted at: (i) pre-implementation; (ii) 1 year post-implementation; and (iii) 3 years post-implementation.

#### Collisions and casualties

These data are continually recorded by the Police Service of Northern Ireland. The database is a collection of all road traffic collisions that resulted in a personal injury and were reported to the police. Collision severity was categorised as fatal, serious and slight. Casualties were categorised as children, elderly, pedestrian, motorcyclist, pedal cyclist. The data were accessed via a data request to the Police Service of Northern Ireland in 2019.

#### Traffic speed and volume

The data were collected by the Department for Infrastructure (Northern Ireland) through commissioned cordon surveys using automatic sensors in 2013/2014 (pre-implementation) to record speed and volume across the proposed 20 mph area. This was repeated in 2017 (1 year post-implementation) and 2019 (3 year post-implementation) across the new 20 mph area, with data being recorded with automatic traffic tube monitors. At each time point, data collection was conducted over a period of 7 days. These data were provided for monitored streets (17 sites pre-implementation and 11 sites post-implementation; 10 sites in common for both periods) in Belfast city centre. The data included: average speed by time of day; average volume per ranges of speed by time of day; and average volume by time of day. The data were accessed via a data request to the Department for Infrastructure (Northern Ireland) in 2019.

### Statistical analyses

All analyses were conducted using STATA 16 SE.^16^

#### Objective 1: to investigate the change in the rate and severity of road traffic collisions and casualties at 1 year and 3 years post-implementation

We calculated the number of traffic collisions in each year between 2014 and 2019. For all the calculations, the ‘before’ period was 1 year in duration (2015; the year before the introduction of the 20 mph speed limits). The ‘after’ period for the city-wide calculations was approximately 1 year and 3 years (ie, in year 3) after implementation of the 20 mph speed limits. We calculated the percentage change in collisions within each site (described above) using 2015 (the year before the introduction of the 20 mph speed limits) as the reference and derived p values for this change using Poisson regression.

We evaluated difference-in-differences in collisions (intervention area vs control areas) across three different time series (baseline, year 1 and year 3).

1. **Intervention site**: streets which implemented 20 mph speed limits
2. **Control site—city centre**: streets in the surrounding city centre area that did not change their speed limits to 20 mph (remaining at 30 mph before and after the 20 mph speed limit implementation) but likely to observe possible spill-over effects
3. **Control site—metropolitan area**: streets in the surrounding metropolitan area (Greater Belfast area) that did not change their speed limits to 20 mph (remaining at 30 mph or 40 mph before and after the 20 mph speed limit implementation)
4. **Matched control**: matched control sites chosen to be similar to the 20 mph intervention area (see Study design above).

Our difference-in-differences models included an interaction term between study area (eg, intervention, matched control) and time period (pre- and post-intervention), which allowed us to test formally for differences in trend. Crucially this method implicitly adjusts for any secular trends that may influence the risk of collision (eg, number of cars registered in the city) under the assumption that these act identically across the intervention and control arms.

#### Objective 2: to investigate the change in traffic speed 1 year and 3 years post-implementation

The mean change in traffic speed before and after the implementation of the 20 mph speed limit intervention was calculated. Similar summaries based on time of day were conducted for traffic speed. We calculated average traffic speed for each street, and across all streets within the 20 mph intervention site. The difference between the mean speed 1 year and 3 years post-implementation of the 20 mph intervention site with data collected previously was calculated. We calculated the median difference in speed across sites and, due to non-normally distributed data, conducted formal hypothesis tests using the Wilcoxon-signed rank test.

#### Objective 3: to investigate the change in traffic volume 1 year and 3 years post-implementation

The mean change in traffic volume (measured in vehicles per hour) before and after the implementation of the 20 mph speed limit intervention was calculated. Similar summaries based on total volume and time of day were conducted for traffic volume. We calculated average traffic volume for each street, and across all streets within the 20 mph intervention site. We calculated the difference in the mean volume 1 year and 3 years post-implementation of the 20 mph intervention site compared with data collected previously. The median difference in volume across sites was calculated and, due to non-normally distributed data, formal hypothesis tests using the Wilcoxon signed rank test was conducted. A pairwise comparison was made between the pre-20 mph and post-20 mph average volume data at the level of site. Analyses of repeated cross-sections of data for traffic volume were conducted at: (i) pre-implementation; (ii) 1 year post-implementation; and (iii) 3 years post-implementation.

## Results

### Objective 1: to investigate the change in the rate and severity of road traffic collisions and casualties at 1 year and 3 years post-implementation

#### Road traffic collisions

Table 1 presents the road traffic collision severity rates for intervention and control sites over time. Overall, road traffic collision data (ie, slight injury and serious injury/fatal) for the intervention site showed reductions of 10% (p=0.47) in the year of implementation (2016), 3% (p=0.82) at year 1 post-implementation (2017), and 15% (p=0.31) at year 3 post-implementation (2019). Reductions in road traffic collision rates were mainly observed for slight injury severity ranging from n=86 at 1 year pre-implementation (2015) to n=72 (−16%, p=0.27) at 3 years post-implementation (2019). Serious injury/fatal collision rates increased from n=2 at 1 year pre-implementation (2015) to n=3 (+50%, p=0.66) at 3 years post-implementation (2019).

**Table 1:** Annual road traffic collision rates by severity rates for intervention and control sites over time

Site/severity	2 years pre-implementation	1 year pre-implementation	Intervention implementation	1 year post-implementation	3 years post-implementation
	2014	2015	2016	2017	2019
Intervention site					
Slight injury	80 (−7%; p=0.64)	86 (Ref)	76 (−12%; p=0.43)	76 (−12%; p=0.43)	72 (−16%; p=0.27)
Serious injury/fatal	4 (+100%; p=0.42)	2 (Ref)	3 (+50%; p=0.66)	9 (+350%; p=0.05)	3 (+50%; p=0.66)
Overall	84 (−5%; p=0.76)	88 (Ref)	79 (−10%; p=0.47)	85 (−3%; p=0.82)	75 (−15%; p=0.31)
Control site (city centre)					
Slight injury	100 (+2%; p=0.89)	98 (Ref)	101 (+3%; p=0.83)	90 (−8%; p=0.56)	106 (+8%; p=0.58)
Serious injury/fatal	6 (+50%; p=0.53)	4 (Ref)	7 (+75%; p=0.37)	12 (+200%; p=0.06)	8 (+100%; p=0.26)
Overall	106 (+4%; p=0.78)	102 (Ref)	108 (+6%; p=0.68)	102 (0%; p=1.00)	114 (+12%; p=0.41)
Control site (metropolitan area)					
Slight injury	1078 (+6%; p=0.19)	1018 (Ref)	1034 (+2%; p=0.72)	974 (−4%; p=0.32)	939 (−8%; p=0.07)
Serious injury/fatal	79 (−11%; p=0.44)	89 (Ref)	102 (+15%; p=0.35)	97 (+9%; p=0.56)	67 (−25%; p=0.08)
Overall	1157 (+5%; p=0.29)	1107 (Ref)	1136 (+3%; p=0.54)	1071 (−3%; p=0.44)	1006 (−9%; p=0.03)
Matched control					
Slight injury	26 (0%; p=1.00)	26 (Ref)	23 (−12%; p=0.67)	25 (−4%; p=0.89)	23 (−12%; p=0.67)
Serious injury/fatal	2 (+100%; p=0.57)	1 (Ref)	1 (0%; p=1.00)	3 (+200%; p=0.34)	1 (0%; p=1.00)
Overall	28 (+4%; p=0.89)	27 (Ref)	24 (−11%; p=0.68)	28 (+4%; p=0.89)	24 (−11%; p=0.68)

Fatal injury is one which causes death less than 30 days after the accident. Serious injury is one which does not cause death but serious injury such as needing to be detained in hospital as an inpatient, fractures, concussion, internal injuries. Slight injury is any injury which is neither ‘fatal’ nor ‘serious’, for example, a sprain, bruise or cut which is not judged to be severe, or slight shock requiring roadside attention.

Ref, reference case.

Increases in slight injury (+8%, p=0.58), serious injury/fatal (+100%, p=0.26) and overall (+12%, p=0.41) for road traffic collisions in the city centre control site at 3 years post-implementation (2019), shown in table 1, were observed. Findings showed decreases for all road traffic collision categories (ie, slight, serious injury/fatal and overall) for both the metropolitan area control site and the matched control site at 3 years post-implementation.

The results from the difference-in-differences analysis showed no statistically significant differences between the intervention site and the three control sites over time (online supplemental file 1). Results showed incidence rate ratios of 1.01 (95% CI 0.81 to 1.26) for the city centre control site, 0.97 (95% CI 0.81 to 1.16) for the metropolitan area, and 0.90 (95% CI 0.64 to 1.27) for the matched control site.

10.1136/jech-2022-219729.supp1 Supplementary data

#### Road traffic casualties

Table 2 presents the results for changes in numbers of casualties over time. Overall, for the intervention site there was a reduction in casualties of 12% (p=0.31) in the year of implementation (2016), 16% (p=0.18) at year 1 post-implementation (2017), and 22% (p=0.06) at year 3 post-implementation (2019). Reductions in casualties were higher for females than males. At 1 year post-implementation (2017) casualties had reduced by 25% (p=0.16) for females in comparison to an 8% reduction (p=0.62) for males. At 3 years post-implementation (2019) reductions remained larger for females at 24% (p=0.19) in comparison to a 20% reduction (p=0.20) for males. Reductions in road traffic casualties were higher for those in the older age categories than in the younger age categories. Results showed reductions of 45% (p=0.23) for those aged 65+ years, 26% (p=0.35) for those aged 50–64 years, and 41% (p=0.05) for those aged 35–49 years. These rates were higher in comparison to a 17% (p=0.76) reduction for the under 16s, a 16% increase (p=0.64) for those aged 16–24 years, and a 21% (p=0.37) reduction for those aged 25–34 years.

**Table 2:** Road traffic casualty rates for intervention and control sites over time

Site/casualty type	2 years pre-implementation	1 year pre-implementation	Intervention implementation	1 year post-implementation	3 years post-implementation
	2014	2015	2016	2017	2019
Intervention site					
Class					
Car	81 (–14%; p=0.33)	94 (Ref)	86 (–9%; p=0.55)	81 (–14%; p=0.33)	69 (–27%; p=0.05)
Pedestrian	27 (–13%; p=0.60)	31 (Ref)	22 (–29%; p=0.22)	22 (–29%; p=0.22)	25 (–19%; p=0.42)
Motorcyclist	–	3 (Ref)	2 (–33%; p=0.66)	4 (+33%; p=0.71)	2 (–33%; p=0.66)
Pedal cyclist	4 (+33%; p=0.71)	3 (Ref)	5 (+67%; p=0.48)	3 (0%; p=1.00)	6 (+100%; p=0.33)
Sex					
Male	60 (–20%; p=0.20)	75 (Ref)	59 (–21%; p=0.17)	69 (–8%; p=0.62)	60 (–20%; p=0.20)
Female	52 (–5%; p=0.77)	55 (Ref)	56 (+2%; p=0.92)	41 (–25%; p=0.16)	42 (–24%; p=0.19)
Age (years)					
Under 16	10 (+67%; p=0.32)	6 (Ref)	13 (+117%; p=0.12)	5 (–17%; p=0.76)	5 (–17%; p=0.76)
16–24	20 (+5%; p=0.87)	19 (Ref)	25 (+32%; p=0.37)	14 (–26%; p=0.39)	22 (+16%; p=0.64)
25–34	21 (–38%; p=0.08)	34 (Ref)	30 (–12%; p=0.62)	24 (–29%; p=0.19)	27 (–21%; p=0.37)
35–49	31 (–16%; p=0.47)	37 (Ref)	27 (–27%; p=0.21)	29 (–22%; p=0.33)	22 (–41%; p=0.05)
50–64	16 (–30%; p=0.27)	23 (Ref)	10 (–57%; p=0.03)	20 (–13%; p=0.65)	17 (–26%; p=0.35)
65+	12 (+9%; p=0.84)	11 (Ref)	10 (–9%; p=0.83)	17 (+55%; p=0.26)	6 (–45%; p=0.23)
Overall	112 (–15%; p=0.22)	131 (Ref)	115 (–12%; p=0.31)	110 (–16%; p=0.18)	102 (–22%; p=0.06)
Control site (city centre)					
Class					
Car	127 (+12%; p=0.37)	113 (Ref)	142 (+26%; p=0.07)	110 (–3%; p=0.84)	113 (0%; p=1.00)
Pedestrian	16 (–24%; p=0.41)	21 (Ref)	21 (0%; p=1.00)	17 (–19%; p=0.52)	28 (+33%; p=0.32)
Motorcyclist	7 (+250%; p=0.12)	2 (Ref)	2 (0%; p=1.00)	7 (+250%; p=0.12)	6 (+200%; p=0.18)
Pedal cyclist	6 (–14%; p=0.78)	7 (Ref)	11 (+57%; p=0.35)	5 (–29%; p=0.57)	13 (+86%; p=0.19)
Sex					
Male	83 (+24%; p=0.19)	67 (Ref)	103 (+54%; p=0.01)	82 (+22%; p=0.22)	75 (+12%; p=0.50)
Female	72 (–5%; p=0.74)	76 (Ref)	73 (–4%; p=0.81)	57 (–25%; p=0.10)	85 (+12%; p=0.48)
Age (years)					
Under 16	10 (+67%; p=0.32)	6 (Ref)	14 (+133%; p=0.08)	6 (0%; p=1.00)	7 (+17%; p=0.78)
16–24	28 (–7%; p=0.79)	30 (Ref)	29 (–3%; p=0.90)	16 (–47%; p=0.04)	27 (–10%; p=0.69)
25–34	38 (+3%; p=0.91)	37 (Ref)	42 (+14%; p=0.57)	38 (+3%; p=0.91)	38 (+3%; p=0.91)
35–49	40 (+8%; p=0.73)	37 (Ref)	52 (+41%; p=0.11)	34 (–8%; p=0.72)	42 (+14%; p=0.57)
50–64	26 (0%; p=1.00)	26 (Ref)	29 (+12%; p=0.69)	32 (+23%; p=0.43)	33 (+27%; p=0.36)
65+	11 (+57%; p=0.35)	7 (Ref)	10 (+43%; p=0.47)	13 (+86%; p=0.19)	12 (+71%; p=0.26)
Overall	156 (+9%; p=0.45)	143 (Ref)	176 (+23%; p=0.07)	139 (–3%; p=0.81)	160 (+12%; p=0.33)
Control site (metropolitan area)					
Class					
Car	1344 (0%; p=0.97)	1346 (Ref)	1256 (–7%; p=0.08)	1168 (–13%; p=0.00)	1138 (–15%; p=0.00)
Pedestrian	218 (–2%; p=0.85)	222 (Ref)	218 (–2%; p=0.85)	211 (–5%; p=0.60)	191 (–14%; p=0.13)
Motorcyclist	55 (0%; p=1.00)	55 (Ref)	58 (+5%; p=0.78)	60 (+9%; p=0.64)	56 (+2%; p=0.92)
Pedal cyclist	105 (+36%; p=0.04)	77 (Ref)	105 (+36%; p=0.04)	103 (+34%; p=0.05)	98 (+27%; p=0.11)
Sex					
Male	948 (+2%; p=0.61)	926 (Ref)	930 (0%; p=0.93)	844 (–9%; p=0.05)	784 (–15%; p=0.00)
Female	774 (0%; p=0.98)	773 (Ref)	707 (–9%; p=0.09)	697 (–10%; p=0.05)	699 (–10%; p=0.05)
Age (years)					
Under 16	198 (+8%; p=0.47)	184 (Ref)	186 (+1%; p=0.92)	169 (–8%; p=0.43)	172 (–7%; p=0.53)
16–24	359 (+7%; p=0.36)	335 (Ref)	278 (–17%; p=0.02)	261 (–22%; p=0.00)	278 (–17%; p=0.02)
25–34	428 (+4%; p=0.58)	412 (Ref)	410 (0%; p=0.94)	373 (–9%; p=0.16)	334 (–19%; p=0.00)
35–49	378 (+1%; p=0.86)	373 (Ref)	396 (+6%; p=0.41)	395 (+6%; p=0.43)	338 (–9%; p=0.19)
50–64	246 (–1%; p=0.93)	248 (Ref)	251 (+1%; p=0.89)	224 (–10%; p=0.27)	256 (+3%; p=0.72)
65+	100 (–24%; p=0.04)	131 (Ref)	113 (–14%; p=0.25)	115 (–12%; p=0.31)	102 (–22%; p=0.06)
Overall	1722 (+1%; p=0.71)	1700 (Ref)	1637 (–4%; p=0.28)	1542 (–9%; p=0.01)	1483 (–13%; p=0.00)
Matched control					
Class					
Car	26 (–7%; p=0.79)	28 (Ref)	26 (–7%; p=0.79)	27 (–4%; p=0.89)	20 (–29%; p=0.25)
Pedestrian	8 (–20%; p=0.64)	10 (Ref)	4 (–60%; p=0.12)	9 (–10%; p=0.82)	6 (–40%; p=0.32)
Motorcyclist	–	4 (Ref)	–	2 (–50%; p=0.42)	4 (0%; p=1.00)
Pedal cyclist	1 (0%; p=1.00)	. (Ref)	–	–	–
Sex					
Male	14 (–22%; p=0.48)	18 (Ref)	13 (–28%; p=0.37)	25 (+39%; p=0.29)	14 (–22%; p=0.48)
Female	21 (–13%; p=0.66)	24 (Ref)	18 (–25%; p=0.36)	13 (–46%; p=0.08)	16 (–33%; p=0.21)
Age (years)					
Under 16	4 (+33%; p=0.71)	3 (Ref)	2 (–33%; p=0.66)	2 (–33%; p=0.66)	2 (–33%; p=0.66)
16–24	7 (+133%; p=0.22)	3 (Ref)	6 (+100%; p=0.33)	5 (+67%; p=0.48)	3 (0%; p=1.00)
25–34	4 (–56%; p=0.18)	9 (Ref)	1 (–89%; p=0.04)	5 (–44%; p=0.29)	6 (–33%; p=0.44)
35–49	8 (–27%; p=0.49)	11 (Ref)	9 (–18%; p=0.66)	11 (0%; p=1.00)	7 (–36%; p=0.35)
50–64	8 (–0%; p=1.00)	8 (Ref)	7 (–13%; p=0.80)	8 (0%; p=1.00)	6 (–25%; p=0.59)
65+	4 (–50%; p=0.26)	8 (Ref)	6 (–25%; p=0.59)	7 (–13%; p=0.80)	6 (–25%; p=0.59)
Overall	35 (–17%; p=0.43)	42 (Ref)	31 (–26%; p=0.20)	38 (–10%; p=0.66)	30 (–29%; p=0.16)

–, missing data; Ref, reference case.

Similar to road traffic collision trends, the control sites demonstrated increases overall for road traffic casualties in the city centre control site at 3 years post-implementation, with decreases for all road traffic casualties for both the metropolitan area control site and the matched control site at 3 years post-implementation.

Difference-in-differences analysis showed no statistically significant differences between the intervention site and the three control sites over time (online supplemental file 2). Results showed incidence rate ratios of 1.02 (95% CI 0.84 to 1.25) for the city centre control site, 0.96 (95% CI 0.82 to 1.12) for the metropolitan area, and 0.84 (95% CI 0.62 to 1.15) for the matched control site (online supplemental file 2).

### Objective 2: to investigate the change in traffic speed 1 year and 3 years post-implementation

In table 3, the pre- and post-implementation datasets were combined to obtain a dataset with paired streets. Some of the streets in the pre-20 mph dataset were removed because there was no corresponding street in the 1 year post-implementation dataset (see online supplemental file 5 for data availability description). The results show that there was little overall change in median traffic speed at year 1 (0.2 mph, 95% CI −0.3 to 2.4, p=0.14) and year 3 post-implementation (0.8 mph, 95% CI −1.5 to 2.5, p=0.17). Mean traffic speed (online supplemental file 3) and mean difference in traffic speed (online supplemental file 4) are visually depicted for year 1 and year 3 post-implementation periods.

**Table 3:** Road traffic speed for intervention streets over time

Time of day	8.00–9.00			12.00–13.00			17.00–18.00			21.00–22.00			Overall*		
Time period	Pre	1 year	3 years	Pre	1 year	3 years	Pre	1 year	3 years	Pre	1 year	3 years	Pre	1 year	3 years
Chichester Street	20.0	20.2 (0.2)	19.9 (−0.1)	17.0	18.0 (1.0)	16.8 (−0.2)	14.1	15.8 (1.7)	15.1 (1.0)	23.1	21.5 (−1.6)	21.3 (−1.8)	19.2	18.9 (−0.3)	17.7 (−1.5)
Donegall Place	14.6	17.1 (2.5)	16.2 (1.6)	11.6	14.2 (2.6)	13.8 (2.2)	11.1	13.7 (2.6)	13.6 (2.5)	15.3	17.8 (2.5)	18.3 (3.0)	14.2	16.5 (2.4)	16.5 (2.3)
Donegall Street	21.5	24.2 (2.7)	24.2 (2.7)	19.1	20.1 (1.0)	20.2 (1.1)	19.4	19.5 (0.1)	20.5 (1.1)	20.3	21.1 (0.8)	21.0 (0.7)	20.2	20.9 (0.7)	21.0 (0.8)
High Street	16.2	20.0 (3.8)	20.6 (4.4)	10.2	17.3 (7.1)	17.3 (7.1)	14.3	16.9 (2.6)	16.6 (2.3)	17.6	18.5 (0.9)	19.3 (1.7)	14.4	17.9 (3.5)	17.8 (3.3)
Howard Street	17.0	19.0 (2.0)	20.7 (3.7)	19.9	16.9 (−3.0)	18.6 (−1.3)	14.7	14.2 (−0.5)	14.2 (−0.5)	18.6	17.9 (−0.7)	20.3 (1.7)	17.2	17.4 (0.2)	18.9 (1.7)
May Street	17.7	20.8 (3.1)	18.6 (0.9)	17.4	19.6 (2.2)	16.5 (−0.9)	15.6	16.3 (0.7)	13.7 (−1.9)	25.7	23.4 (−2.3)	20.6 (−5.1)	19.9	20.1 (0.2)	17.4 (−2.6)
North Street	20.4	^–^	19.7 (−0.7)	15.8	–	17.6 (1.8)	16.6	–	17.7 (1.1)	22.7	–	19.8 (−2.9)	18.7	–	18.5 (−0.2)
Queen Street	18.0	16.0 (−2.0)	20.2 (2.2)	17.1	14.5 (−2.6)	16.6 (−0.5)	16.9	15.0 (−1.9)	16.2 (−0.7)	18.3	16.7 (−1.6)	20.4 (2.1)	17.5	15.1 (−2.3)	18.0 (0.5)
Royal Avenue	14.6	17.9 (3.3)	16.0 (1.4)	11.6	16.2 (4.6)	15.3 (3.7)	11.1	15.5 (4.4)	15.1 (4.0)	15.3	18.6 (3.3)	16.2 (0.9)	13.2	17.1 (4.0)	15.6 (2.5)
Wellington Place	16.2	16.5 (0.3)	20.9 (4.7)	15.0	14.8 (−0.2)	19.1 (4.1)	14.6	14.8 (0.2)	18.5 (3.9)	19.7	18.4 (−1.3)	20.7 (1.0)	16.9	16.4 (−0.4)	19.9 (3.1)
York Street	21.6	23.8 (2.2)	20.8 (−0.8)	21.1	21.8 (0.7)	19.5 (−1.6)	19.6	19.9 (0.3)	18.7 (−0.9)	24.4	23.3 (−1.1)	21.3 (−3.1)	21.8	21.8 (−0.0)	19.8 (−2.0)
Overall*†	18.2	20.0 (1.8)	20.4 (2.1)	17.1	17.9 (0.8)	18.1 (1.0)	15.8	16.5 (0.7)	16.7 (0.9)	20.7	20.1 (−0.6)	20.5 (−0.3)	18.2	18.6 (0.4)	18.8 (0.6)
Overall (unweighted)†	17.7	19.6 (1.8)	19.8 (2.1)	16.0	17.3 (1.3)	17.4 (1.4)	15.1	16.1 (1.0)	16.2 (1.1)	19.8	19.7 (−0.1)	19.9 (0.1)	18.9	19.4 (0.5)	19.7 (0.8)
Median Difference		2.3 (0.3 to 3.1)	1.6 (−0.1 to 3.7)		1.0 (−0.2 to 2.6)	1.1 (−0.9 to 3.7)		0.5 (0.1 to 2.6)	1.1 (−0.7 to 2.5)		−0.9 (−1.6 to 0.9)	0.9 (−2.9 to 1.7)		0.2 (−0.3 to 2.4)	0.8 (−1.5 to 2.5)
P value		P=0.09	P=0.01		P=0.13	P=0.08		P=0.04	P=0.03		P=0.58	P=0.97		P=0.14	P=0.17

–, Missing data; (.) change in speed from pre-implementation.

*Overall speed is a weighted average using pre-study volume.

†Restricted to sites with data recorded at all time points.

### Objective 3: to investigate the change in traffic volume 1 year and 3 years post-implementation

Table 4 presents the results for road traffic volume for intervention streets at pre-implementation, year 1 and year 3 post-implementation. For traffic volume, a statistically significant decrease in 57 vehicles per week (95% CI –162 to −14, p<0.00) was observed when considering pre- and post-implementation (1 year) comparisons for matched streets. Decreases in traffic volume were also observed when comparing all sites pre- and 3 years post-implementation (−71 vehicles per week, 95% CI −213 to 1, p=0.05).

**Table 4:** Road traffic volume for intervention streets over time

Street	8.00–9.00			12.00–13.00			17.00–18.00			21.00–22.00			Overall*		
	**Pre**	**1 year**	**3 years**	**Pre**	**1 year**	**3 years**	**Pre**	**1 year**	**3 years**	**Pre**	**1 year**	**3 years**	**Pre**	**1 year**	**3 years**
Chichester Street	531	416 (−115.1)	382 (−149.0)	666	577 (−88.9)	429 (−237.0)	613	689 (75.7)	408 (−205.0)	364	396 (31.9)	300 (−64.0)	424	397 (−27.0)	320 (−104.2)
Donegall Place	115	98 (−16.7)	47 (−68.0)	130	108 (−22.4)	59 −71.0)	123	131 (8.0)	75 (−48.0)	107	102 (−4.6)	74 (−33.0)	100	99 (−0.8)	63 (−37.2)
Donegall Street	461	124 (−337.0)	125 (−336.5)	560	220 (−339.8)	222 (−338.0)	626	267 (−359.2)	259 (−367.5)	368	154 (−213.7)	168 (−200.5)	378	161 (−216.7)	165 (−212.6)
Howard Street	678	511 (−166.6)	486 (−192.0)	687	554 (−133.0)	592 (−95.0)	657	652 (−5.3)	528 (−129.0)	398	414 (15.6)	446 (48.0)	454	406 (−48.3)	420 (−34.2)
May Street	686	522 (−164.4)	509 (−177.0)	589	489 (−100.1)	553 (−36.0)	487	467 (−20.4)	574 (87.0)	297	284 (−12.7)	362 (65.0)	388	322 (−65.9)	389 (1.0)
North Street	441	144 (−296.9)	55 (−386.0)	557	178 (−379.1)	97 (−460.0)	646	299 (−347.4)	112 (−534.0)	289	125 (−164.4)	57 (−232.0)	335	142 (−193.2)	61 (−274.1)
Queen Street	94	94 (0.3)	117 (22.5)	141	132 (−8.6)	222 (81.0)	125	119 (−6.0)	288 (162.5)	52	53 (1.0)	132 (80.0)	74	74 (0.5)	147 (73.0)
Royal Avenue	170	93 −77.4)	271 (101.0)	148	126 (−22.5)	335 (187.3)	165	149 (−15.6)	339 (174.3)	74	63 (−11.2)	232 (158.0)	96	82 (−14.2)	234 (137.8)
Wellington Place	722	479 (−242.9)	95 (−627.5)	842	586 (−256.4)	162 (−680.5)	697	564 (−132.9)	213 (−484.5)	484	427 (−56.9)	90 (−394.5)	538	415 (−123.1)	109 (−428.5)
York Street	457	131 (−326.3)	94 (−363.0)	561	211 (−349.9)	166 (−395.0)	533	309 (−223.6)	261 (−272.5)	212	97 (−115.5)	78 (−134.5)	305	143 (−162.2)	116 (−189.8)
Overall*	4355	2612 (−1743.0)	2180 (−2175.5)	4881	3180 (−1700.6)	2837 (−2044.3)	4672	3645 (−1026.6)	3055 (−1616.8)	2645	2115 (−530.4)	1938 (−707.5)	3092	2241 (−850.8)	1996 (−1095.5)
Median Difference		−166 (−297 to –77)	−185 (−363 to –68)		−117 (−340 to –23)	−166 (−395 to –36)		−18 (−224 to –5)	−167 (−368 to 87)		−12 (−116 to 1)	−49 (−201 to 65)		−57 (−162 to –14)	−71 (−213 to 1)
P value		P<0.00	P=0.01		P<0.00	P=0.03		P=0.03	P=0.05		P=0.06	P=0.18		P<0.00	P=0.05

(.) Change in volume from pre-implementation.

*Restricted to sites with data recorded at all time points.

Particular reductions in traffic volume were observed for the morning commuting time (8.00–9.00 am). A decrease in 166 vehicles per week (95% CI –297 to −77, p<0.00) was observed when considering pre- and post-implementation (1 year) comparisons for matched streets. A statistically significant decrease in traffic volume was also observed when comparing all sites pre- and 3 years post-implementation (−185 vehicles per week, 95% CI −363 to –68, p=0.01).

## Discussion

### Summary of key findings

We investigated the effectiveness of a 20 mph speed limit intervention on road traffic collisions and casualties, speed and volume at 1 year and 3 years post-implementation in Belfast city centre. This city centre implementation of an intervention involved the introduction of 20 mph signage, with estimated implementation costs of approximately £10 000. In summary, our findings suggest little effect for reduction in road traffic collisions, casualties and speed when a 20 mph speed limit intervention is implemented in a city centre. We did observe significant reductions in road traffic volume. The increases observed for slight injury, serious injury/fatal and overall for road traffic collisions in the city centre control site may be due to spill-over effects from the neighbouring intervention streets. Although not statistically significant (at the 5% level), any reduction in the number of fatalities is of public health importance.

### Compare/contrast with the literature

Our findings add to the current limited body of research^17^ evaluating the effectiveness of 20 mph speed limit interventions. This study is one of the first studies to evaluate the impact of 20 mph speed limits 3 years post-implementation.

Our results are similar to the findings from a pilot scheme in Portsmouth,^18^ which demonstrated an overall reduction in traffic speed of between 0.9 mph and 1.9 mph on roads where 20 mph limits were implemented. An average reduction of 6.3 mph was seen on roads that were characterised by speeds of over 24 mph before the lower limits were introduced. The city also showed a 22% reduction in reported road traffic casualties where 20 mph restrictions had been introduced.

Much larger effects have been demonstrated when 20 mph speed limit interventions are implemented city-wide in comparison to city centre. The example in Bristol^19^ has been used by many local authorities to support the introduction of 20 mph limits. Research evaluating the effectiveness of the city-wide 20 mph speed limit intervention in Bristol found statistically significant reductions in average traffic speeds of 2.7 mph, and a reduction in the number of fatal, serious and slight injuries from road traffic collisions, equating to estimated cost savings of over £15 million per year.^19^ Similarly, in a city-wide 20 mph speed limit intervention in Edinburgh,^17 20^ evidence highlighted that, following the implementation of the 20 mph policy, a further reduction in collision rates occurred. Specifically, the average number of collisions per month in Edinburgh in 1997 was 165, while in 2019 this number fell to 64.^17 20^

In contrast, Manchester City Council^21^ withdrew funding for the 20 mph speed limits based mainly on the fact that the decrease in the number of road traffic collisions in 20 mph speed limit areas was not as great as it was on the 30 mph roads. Atkins and Maher^22^ suggested that the reasons for the decline resulted from the closure of public counters and phone lines at some police stations, making it more difficult for the public to report collisions, which have not been attended by the police. Second, it was also suggested that increasing levels of congestion, partly linked to roadworks associated with development and construction sites, may have reduced speeds and collisions on 30 mph roads.

The findings from our current study are in line with qualitative research conducted in Belfast with members of the public.^23^ Cleland *et al* 23 reported that members of the public perceived little change in traffic speed following the implementation of the 20 mph speed limit intervention in Belfast. Four unintended pathways were identified from the focus groups that included the mechanism of ‘no change in driving speed’.^23^ These pathways related to: the lack of awareness of the 20 mph speed intervention; the perception that there was no need for the reduced speed limits within the city centre which meant drivers did not have to self-regulate their speed; and the lack of enforcement which resulted in participants having no fear of punishment for driving above the speed limit.^23^

We did observe significant reductions in road traffic volume. The 20 mph speed limit may have reduced traffic volume due to changes to alternative routes or change in travel mode. However, we do not have data that provide evidence of traffic displacement or change in travel mode to make a substantive statement on the reason for reduction in traffic volume.

### Recommendations for future policy and practice

Policymakers considering implementing 20 mph speed limit interventions should consider the fidelity, context and scale of implementation. Our qualitative evaluation^23^ highlights that some drivers were unaware of the 20 mph speed limit intervention and that enforcement was unlikely (fidelity). Future 20 mph speed limit interventions should consider the importance of awareness/education and enforcement elements.^23^ In general, the mean speed pre-implementation across most streets was below 20 mph, which questions whether a 20 mph speed limit intervention was necessary in Belfast city centre (context). The intervention was implemented at the city centre scale (only 76 streets) in comparison to the recent city-wide intervention in Edinburgh which showed significant reductions in road traffic speed, collisions and casualties.^17 20^ Large scale implementation of 20 mph speed limit interventions may be an important factor for effectiveness (scale).

Previous research has suggested that 20 mph speed limit interventions should be supplemented with other interventions such as driver training, social marketing, community engagement, closed-circuit television (CCTV), in-car interventions, community interventions (eg, speed watch), and police communications.^23^ Such success may then have the capacity to facilitate an ambitious culture change that shifts populations away from the car-dominant paradigm and help us recognise that 20 mph speed limits are not simply a road safety intervention, but instead part of the fundamental reset of the way we choose our life priorities—people before cars.

### Recommendations for future research

These results pose several scientific and real-world implementation challenges,^24^ including how to interpret conflicting outcomes.^25^ Other methods from a systems thinking perspective, such as contribution analysis with process tracing^26^ and ripple effects mapping,^27^ and adopting a Bayesian decision framework,^28^ could help disentangle multiple outcomes.

Although the relationship can be complex, transport and health are inextricably linked. Traffic interventions have many knock-on effects, and it is clear that 20 mph speed zones and limits may be more than just a road safety measure. Therefore, when evaluating their effectiveness as a public health intervention it is important to also consider these wider outcomes. Additionally, the incorporation of prior knowledge, such as estimates from Elvik’s models and from relevant systematic reviews, within a Bayesian framework^29^ will allow for a broader modelling approach to the evaluation of the impact of 20 mph speed limit interventions on road traffic collisions.

### Strengths and limitations

The analysis cannot determine how much of the observed changes can be attributed to the intervention (rather than other events or interventions within the timeframe under analysis). There are a number of covariates that can potentially influence the outcomes: other activities occurring in the city centre, including less than optimal implementation, the fluctuating changes in fuel prices or changes in public transit systems, and the large differences in collisions/casualties observed in other areas of the city (eg, metropolitan area), suggest that other factors beyond the intervention were important. The effectiveness of any complex intervention is not solely reliant on the activities and change that is anticipated. Effectiveness is dependent on the systems into which an intervention is implemented, combined with the social, cultural, political and geographical context.

However, the theory-based approach that we utilised in our evaluation^6^ and the pre-stated protocol mean our results add weight to the evidence base for 20 mph limits leading to impacts at 1 year and 3 years post-implementation. The nature of this study, being a natural experiment evaluation, presents researchers with the need to acknowledge confounding variables and make adjustments in analyses where possible.^30^

## Conclusions

Our findings showed that a city centre 20 mph intervention had little impact on long-term outcomes including road traffic collisions, casualties and speed, except for a reduction in traffic volume. Future 20 mph speed limit interventions should consider the fidelity, context and scale of implementation.

## Data Availability

All data relevant to the study are included in the article or uploaded as supplementary information.
