# Supplementary material for: Investigating the impact of a 20 miles per hour speed limit intervention on road traffic collisions, casualties, speed and volume in Belfast, UK: 3 year follow-up outcomes of a natural experiment
Source: J Epidemiol Community Health. 2022 Nov 15;77(1):17–25. doi: 10.1136/jech-2022-219729 (PMC9763225; doi:10.1136/jech-2022-219729)
Supplement: Supplementary data [file jech-2022-219729supp001.pdf]

Supplementary File:

Supplementary File 1: Results for the difference-in-difference analysis over time for road traffic collisions

Site	IRR (95% CI)
Control site (City centre)	1.01 (0.81,1.26)
Control site (Metropolitan area)	0.97 (0.81,1.16)
Matched control	0.90 (0.64,1.27)

CI: Confidence Interval; IRR: Incidence Rate Ratio.

Supplementary File 2: Results for the difference-in-difference analysis over time for road traffic casualties

Site	IRR (95% CI)
Control site (City centre)	1.02 (0.84,1.25)
Control site (Metropolitan area)	0.96 (0.82,1.12)
Matched control	0.84 (0.62,1.15)

CI: Confidence Interval; IRR: Incidence Rate Ratio.

Supplementary File 3: Mean traffic speed over 24 hour period at pre-implementation, 1 and 3 years post-implementation

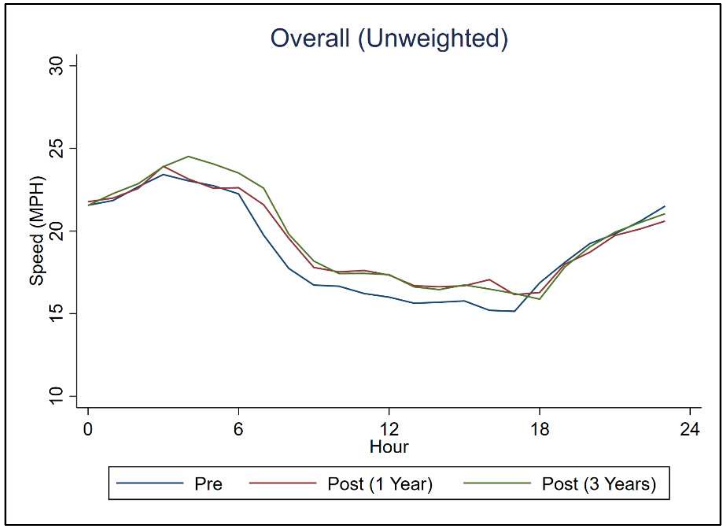

Supplementary File 4: Mean difference in traffic speed at 1 and 3 years post-implementation

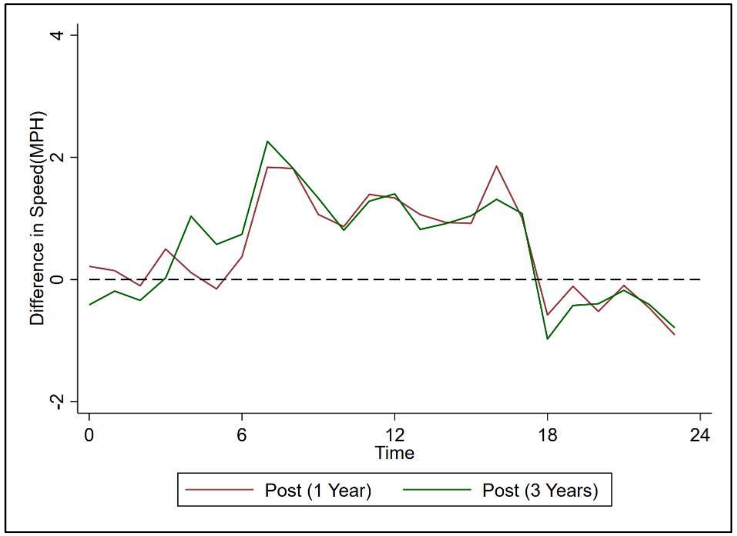

Supplementary File 5: Data Availability

The table below shows the data available for each intervention street for traffic speed and volume pre-implementation, year 1 and year 3 post-implementation periods.

**Street**	**Speed**			**Volume**		
	**Pre-implementation**	**1 year post-implementation**	**3 years post-implementation**	**Pre-implementation**	**1 year post-implementation**	**3 years post-implementation**
Chichester Street	✓	✓	✓	✓	✓	✓
Donegall Place	✓	✓	✓	✓	✓	✓
Donegall Street	✓	✓	✓	✓	✓	✓
Howard Street	✓	✓	✓	✓	✓	✓
May Street	✓	✓	✓	✓	✓	✓
Queen Street	✓	✓	✓	✓	✓	✓
Royal Avenue	✓	✓	✓	✓	✓	✓
Wellington Place	✓	✓	✓	✓	✓	✓
York Street	✓	✓	✓	✓	✓	✓
High Street	✓	✓	✓	X	✓	✓
North Street	✓	X	✓	✓	✓	✓
College Ave Millifield	✓	X	X	✓	X	X
Dunbar Link	✓	X	X	✓	X	X
Royal Ave Control	✓	X	X	✓	X	X
Overall (Unweighted)	✓	✓	✓	X	X	X
College Ave	✓	X	X	X	X	X
College Ave two	✓	X	X	X	X	X
Victoria St	✓	X	X	X	X	X
Victoria St Carriageway	✓	X	X	X	X	X
Victoria St at Mboro	✓	X	X	X	X	X
College Ave City bounc	X	X	X	✓	X	X
College Ave College Square	X	X	X	✓	X	X
Overall	✓	✓	✓	✓	✓	✓
